# The Influence of Environmental Factors on Site Selection Augment Breeding Success in Honey Bees: An Insight of Honey Bee Genetic Resource Conservation

**DOI:** 10.3390/biology13060444

**Published:** 2024-06-18

**Authors:** Peter Njukang Akongte, Bo-Sun Park, Minwoong Son, Chang-hoon Lee, Daegeun Oh, Yong-Soo Choi, Dongwon Kim

**Affiliations:** 1 Department of Agricultural Biology, National Institute of Agricultural Science, Wanju 55365, Republic of Korea; akongtepeter@korea.kr (P.N.A.); crambinae@korea.kr (B.-S.P.); sonmiou807@korea.kr (M.S.); lch0787@korea.kr (C.-h.L.); dheorms2@korea.kr (D.O.); beechoi@korea.kr (Y.-S.C.); 2 Institute of Agricultural Research for Development (IRAD), Buea PMB 25, Cameroon

**Keywords:** controlled breeding, mating success, honey bees, mating station

## Abstract

**Simple Summary:**

Addressing challenges of controlled breeding in honey bees at natural mating stations is important in controlling and maintaining valuable traits of biological and economic interest. Basically, artificial insemination and natural mating are the two reproductive techniques used by beekeepers in maintaining honey bees’ genetic resources. However, little is known on the various sources of variation affecting breeding success at different mating stations. Our study provides an insight, addressing key questions to improve mating success rate of controlled breeding. Selecting mating stations with the maximum cost benefit return is a motivating tool in maintaining valuable genetic materials of honey bees. Our results demonstrated that breeding success of natural mating at controlled mating stations is influenced by the environmental conditions of the area. Mating success rate was high in mating stations with a high percentage of bare land, coniferous forests, deciduous forests, fields, and mixed forests. We suggest that mating stations with mixed forest and fields are potential sites for successful breeding and maintenance of honey bees’ genetic resources.

**Abstract:**

Honey bee reproductive behavior involves a complicated mating system that embodies a number of factors, including environmental and human-induced factors. Controlled breeding in isolated mating stations is a prerequisite to maintain the genetic resources of honey bees through natural mating. The concept of controlled mating is a challenge in most beekeeping operations due to its low mating success rate. Therefore, a detailed investigation into the suitability of isolated mating stations is of interest. Thus, we bred two subspecies of honey bees (*Apis cerana koreana* and *Apis mellifera* L.) in isolated mating stations (island) from 2021 to 2023 and in an open breeding station in 2023. Our results demonstrate that the highest percentage of the mating success rate in isolated mating stations was recorded in the Wido Island, which had the highest percentage of bare land, coniferous forests, deciduous forests, fields, and mixed forests. The mating success rate was higher in the summer and spring for *A. cerana* and *A. mellifera*, respectively. The mating success rate was higher in open mating compared to controlled mating (Island) and did not vary between pure-breeding and cross-breeding lines. Our findings suggested that mating stations with mixed forest and fields are potential sites for the successful breeding of honey bees.

## 1. Introduction

Reproductive success is used to depict breeding success in animals. The common goal of reproduction in animals is the production of offspring, though the process may vary from species to species. In honey bees, reproductive behavior involves a complicated mating system that embodies a number of factors, including environmental and human-induced factors. This behavior is associated with a complex polyandrous mating system [1] where queen bees mate naturally with 15–20 drones in a single flight [2] and in two flights [3], and in a drone congregation area consisting of about 10,000–30,000 drones [2]. It is well known that queen bees are unique [1,4] and are the most important in a honey bee colony due to their responsibility of laying eggs and keeping the colony alive.

Beekeepers depend solely on the quality of their queen bees for colony development and the production of hive products. However, biotic and abiotic factors are known to disrupt the quality of queen bees and consequently affect their reproductive success, some of which include age of the transferred larvae; origin of the larvae; the number of young worker bees; the food presence of starter and finisher colonies; the amount of mated queen bees with a sufficient number of drone bees [5]; and queen mandibular pheromones [6]. In recent years, most beekeepers have neglected the aspect of reproductive success that relies on drone quality, which is one of the key components of honey bee breeding. Abiotic factors could alter drone quality and breeding success, though little is known on these topics. For instance, Rhodes [7] reported that drone sexual maturity is affected by climate, nutrition, and other environmental factors. Several studies have addressed pertinent aspects of reproduction in honey bees while focusing on queen quality [8,9,10,11,12], drone quality [13,14,15,16,17,18,19], and environmental factors [20,21].

Modern-era animal breeding was first described by Sir Robert Bakewell (1725–1795) and later spread to the rest of Europe and North America [22,23]. Natural mating in animals has been categorized into free mating and controlled mating systems. The genetic material of an organism cannot be maintained through open mating but can be achieved by practicing controlled mating. However, the concept of controlled mating in honey bees was first established by attempting the use of isolated mating stations, which were unsuccessful, in Switzerland [24]. Recently, isolated mating stations have become successfully developed and popular in Central European honey bee breeding [25]. Again, the development of artificial insemination techniques for honey bee queens (controlled breeding) in the late nineteen century is now a practical tool in economic bee breeding [14]. Recent studies have revealed that the breeding success rate of naturally mated queen bees is far lower for controlled mating (47%) compared to uncontrolled mating (99%) [26]. The low success rate of controlled mating could be an inhibiting factor in maintaining large amounts of genetic resources, especially in developing countries where controlled mating is difficult to achieve [27,28]. The unwanted low percentage of breeding success for controlled mating could be associated with both environmental and human-induced factors. Although some limiting factors (the age of transferred larvae, origin of larvae, the number of young worker bees) of natural mating in honey bees could be under control, the availability of nectar and pollen sources are essential for the growth, development, and survival of honey bee colonies [29]. Therefore, Decourty et al. [30] suggested that further agriculture and landscape changes might alter bee foraging areas and consequently lead to a deficiency in their sources of food, which may have a negative effect on honey bee populations. This is because honey bees depend on transformed nectar and pollen in the form of honey and beebread, respectively [31]. 

In honey bees, breeding success of naturally mated queen bees at controlled mating stations could be linked to the environmental conditions of an area. The emergence of controlled mating in honey bees at selected mating stations is important in controlling and maintaining valuable traits of biological and economic interest. However, there is not much information or literature studies on the various sources of variation affecting breeding success at different mating stations. According to the literature, some studies that have addressed the challenges related to honey bee reproductive success include research on the characteristics of rearing queen bees (*Apis mellifera* L.) in queenright and queenless colonies [32]; observations of the mating behavior of honey bee (*Apis mellifera* L.) queens using radiofrequency identification (RFID);factors influencing the duration and frequency of nuptial flights [21]; the effects of rearing periods on some reproductive characteristics of Caucasian (*Apis mellifera caucasica*) queen bees [33]; breeding success and genetic diversity in honey bees [34]; the importance of controlled mating in honey bee breeding [26]; factors affecting the reproductive health of honey bee (*Apis mellifera*) drones [18]; the mating frequency of *Apis mellifera jemenitica* under desert conditions of Saudi Arabia [35]; and factors influencing the reproductive ability of male bees [36]. Today, few studies are being conducted in this aspect perhaps because of lack of potential mating stations in some regions of the world. For instance, controlled mating could be an inhibitory factor in successful animal breeding in developing countries [27,37]. To our knowledge, many challenges of controlled breeding in honey bees at natural mating stations remain undefined. Our study provides an insight into these challenges, addressing key questions to improve the mating success rate of controlled breeding. Selecting mating stations with the maximum cost benefit return is a motivating tool to increase the probability of maintaining valuable genetic materials in honey bees. It is necessary to identify suitable isolated mating stations to better conserve the genetic material of honey bees of particular interest. This study is a gateway to improve the breeding success rate of controlled breeding at natural mating stations, which is beneficial to beekeepers in maintaining and conserving honey bee genetic resources of interest.

## 2. Materials and Methods

### 2.1. Location of Mating Stations and Determination of Landscape Factors

**Figure 1:**
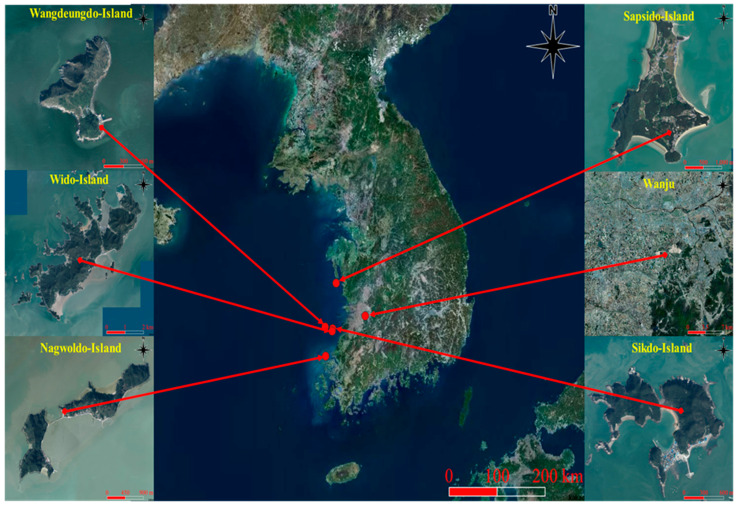
Geographical location of the selected breeding stations within the Korean peninsula. Arrows directly depict the island station within the peninsula.

Mating was conducted in the mainland and islands for closed and open breeding, respectively. The mainland station was located at the experimental apiary (35°49′49.8″ N, 127°02′17.9″ E) of the honey bee breeding laboratory of the National Institute of Agricultural Sciences, Rural Development Administration, Wanju, Republic of Korea (Figure 1). The presence of many small and large islands surrounding three sides of the Republic of Korea has made the country an important region to practice closed or controlled breeding by creating isolated mating stations. Five islands (breeding or mating stations) were selected based on a minimum distance of 31 km to the mainland or nearest coastline. The breeding stations included Wido Island (35°35′26.9″ N, 126°16′46.8″ E), Sapsido Island (36.341° N, 126.357° E), Wangdeungdo Island (35.6572° N, 126.1106° E), Nagwoldo Island (35.2026° N, 126.1337° E), and Sikdo Island (35.6303° N, 126.2877° E). The available landscape factors of the breeding stations were determined with the use of QGIS 3.28 [38]. The factors were classified into bare land, coniferous forests, deciduous forests, facilities, fields, grasslands, mixed forests, rice fields, roads, water, and wetlands, as per breeding station. Other environmental factors (weather factors) including temperature, humidity, cloud cover, and amount of rainfall were not evaluated directly; rather, they were considered based on the seasons of the year [39]. The anomalies of these factors can only be noticeable after many years of observation and thus could not alter the scope of our study within the given time interval. According to the meteorological administration of Korea [40], the mean annual temperature, humidity, and precipitation in the selected islands and their vicinities have not experienced a significant variation between 2021 and 2023 (Table 1).

By implication, these factors vary with season and were similar in the different stations [40].

### 2.2. Drone and Queen Rearing for Breeding

We selected 3–4 strong queenright colonies per breeding line with desired characteristics for drone rearing. In *Apis mellifera*, two drone combs were inserted in-between brood combs of each colony for the queen to lay unfertilized eggs. Two days later, the combs were checked for the presence of eggs and recorded as day 1. In *Apis cerana*, we adopted two methods for drone rearing. Firstly, the bottom section of the brood comb with a few drones was cut off and re-inserted into the colony for the worker bees to build drone cells. Five days later, the comb area was checked for the presence of eggs. Secondly, a foundation comb of *Apis mellifera* base was inserted into the selected drone colony in *Apis cerana* for the workers to construct drone cells. After constructing the drone comb, the comb was removed and re-inserted in-between brood combs for the queen to lay unfertilized eggs. The presence of eggs was checked daily. Regarding the developmental stages of drone honey bees, drones emerge within 24 days [18,41,42] and gain sexual maturity between 6 and 16 days after emergence [18]. Drone colonies were fed more of pollen patty to improve on drone quality (weight and body size) for efficient copulation and sperm viability [36]. Drone colonies were taken to mating stations 2–5 days before drone emergence. Two strong queenright colonies of the desired breeding lines were selected for queen rearing. The age of grafted larvae is a prominent factor that influences the mating behavior of honey bee queens [8]. First-instar (12–20 hold) larvae were transferred into artificial queen cell cups using a Chinese grafting tool [43]. The standard method for rearing *Apis mellifera* queens [44] was adopted and used for rearing both *A. cerana* and *A. mellifera* queens as detailed by Akongte et al. [12]. After 10 days of queen rearing (two days prior to queen emergence), pupae were transferred in queen cells into mating nuclei and placed at mating stations. Queen rearing colonies were fed more sugar syrup. 

### 2.3. Description of Breeding Colonies and Breeding Lines

In our experimental apiary, we grafted larvae for breeding based on the breeding line and the type of breeding. We carried out pure-breeding and crossbreeding at selected breeding stations. In pure-breeding, drone bees and queen bees were raised from the same breeding line. For instance, in the “R” breeding line, we raised drone and queen bees from line R and labeled the mating hives as RR (Figure S1). In crossbreeding, we raised drone and queen bees from two different breeding lines. For example, when drone bees were from line R and queen bees from line X, we labeled the mating hives as XR; when the drone bees were from line X and queen bees from line R, we labeled the mating hives as RX (Figure S1). In each breeding station, we placed drone colonies from one breeding line throughout the breeding period and not a mixture of drones. However, in the case of queen bees, the pure-breeding queen bees and the crossbreeding queen bees could be placed in the same mating station and labeled differently. In this case, the emerging queen bees were marked with different color markers for reliability and easy identification. We bred two pure-breeding lines of *A. cerana* (RR and XX) and crossbreeding lines to form two crossbred lines (RX and XR). In *A. mellifera*, we bred five pure lines (CC, VV, DD, AA and FF) and seven crossbred lines (AC, FD, VF, CD, VD, ACD and AD). 

### 2.4. Preparation, Transportation, and Placement of Drone Colonies and Mating Hives at Mating Stations

We adopted the following methods in preparing drones and mating hives for mating stations: (1): All emerged drones were destroyed from the drone colonies before transporting to the mating stations. (2): The drone colonies must consist of two food combs (honey comb), drone combs (the number of drones depends on the quantity needed by the breeder), brood combs, and a feeder. (3): The combs were fixed by placing a rod horizontally at the front of the feeder to prevent displacement during transportation. (4): The colony was covered with a propolis net beneath the lid to allow air circulation and the entire colony was fastened with a rope before transporting. The mating hives to be transported to the mating stations included a food comb, one brood comb with worker bees, and a feeder. All drone cells, drone broods, and drone bees were destroyed. The combs were fixed by placing a rod horizontally at the front of the feeder to prevent movement during transportation. A special mating hive was prepared with two brood combs consisting only of worker bees for transporting queen cells in-between the sealed brood combs. All mating hives were fastened with a rope before transportation. The drone colonies and mating hives were prepared and kept overnight at the experimental in-land apiary before transportation to the mating stations. In the early hours of the following day, all the colonies were moved to their respective mating stations by car and by ship [45]. At mating stations, we inserted 2 queen cells into each mating hive and opened the hive entrance a few minutes later. Hives were placed in a scattered manner with entrances facing different directions (Figure S2). At the mating stations, all colonies were covered with a propolis net and a piece of cloth beneath the lid.

### 2.5. Evaluation of Mating Success Rate and Transportation of Mating Hives to the Mainland

We evaluated the mating success rate based on the ability of the queen bee to lay fertilized eggs after the nuptial flight [46]. Two days after placing the mating hives at the mating stations, the queen bees were expected to emerge. Two weeks later, the presence of eggs was checked and recorded. Twenty-eight days after placing the colonies at the mating stations, the presence of sealed broods was checked. According to Meyer-Rochow and Jung [47], the presence of fertilized eggs and worker broods is a sign of successful mating. Within this period, all mating hives were moved to the in-land apiaries where successfully mated colonies were labeled and kept, while unsuccessfully mated colonies were discarded. Successfully mated colonies were labeled based on the breeding year and the breeding line. The breeding process was conducted in three consecutive years (2021, 2022, and 2023). The subspecies of *Apis cerana* used in this study is *Apis cerana koreana* [48].

### 2.6. Data Analysis

Landscape factors were analyzed using QGIS 3.28 [38]. The relationship between various environmental factors and the mating success rate was determined using Spearman’s correlation. One-way analysis of variance (ANOVA) was used to compare the means of more than two groups, followed by the Tukey’s (HSD) post hoc test for multiple pairwise comparisons of variance. A two-tailed Student’s *t*-test was used to compare the means of the two groups. The non-parametric Kruskal–Wallis test, followed by multiple pairwise comparisons of variance using Dunn’s procedure, was used to compare the means of more than two groups that were not normally distributed. The XLSTAT statistical software version 2007.8.04 was used to conduct the analysis, with levels of significance set at 5%.

## 3. Results

### 3.1. Characterization of Landscape Factors of the Breeding Stations

**Figure 2:**
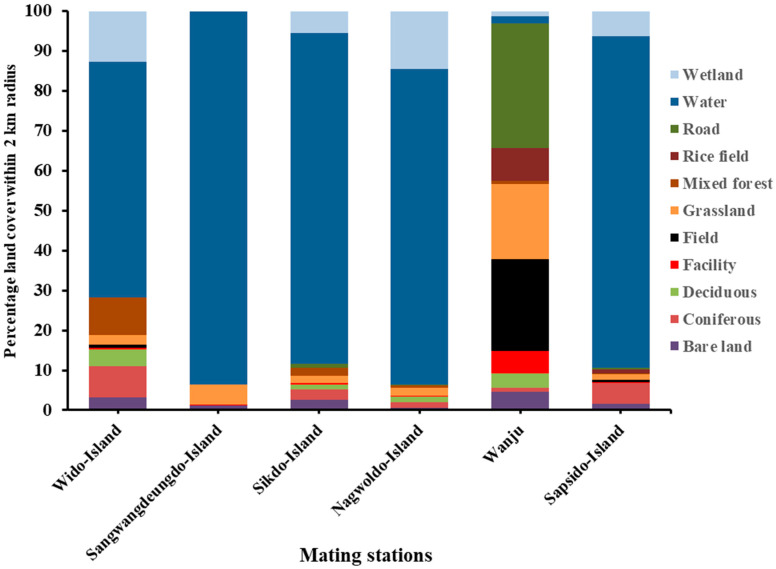
Percentage of land covers within 2 km radius of each mating station.

Landscape factors within a 2 km radius of the respective breeding stations were analyzed (Figure 2).

The land covers of the breeding stations were evaluated based on the following characteristics: bare land, fields, coniferous land, deciduous land, facilities, fields, grassland, mixed forests, rice fields, water, and wetland (Figure 2). Among the five islands selected for breeding, the highest percentage of bare land, coniferous, deciduous, field, and mixed forest was recorded from the WiddoIsland, while the highest percentage of grassland and wetland was recorded from SangwangdeungdoIsland and NagwoldoIsland, respectively (Figure 2). The least percentage of mixed forest was recorded in the SangwangdeungdoIsland and the SapsidoIsland (0% and 0.17%, respectively). In our mainland apiary (mixed breeding station on Wanju Island), we recorded a low percentage of coniferous land and water (1.04% and 1.84%, respectively) and the highest percentage of fields and grasslands (23.07% and 18.69%, respectively).

### 3.2. Seasonal and Annual Variation in the Mating Success Rate of Honey Bees

**Figure 3:**
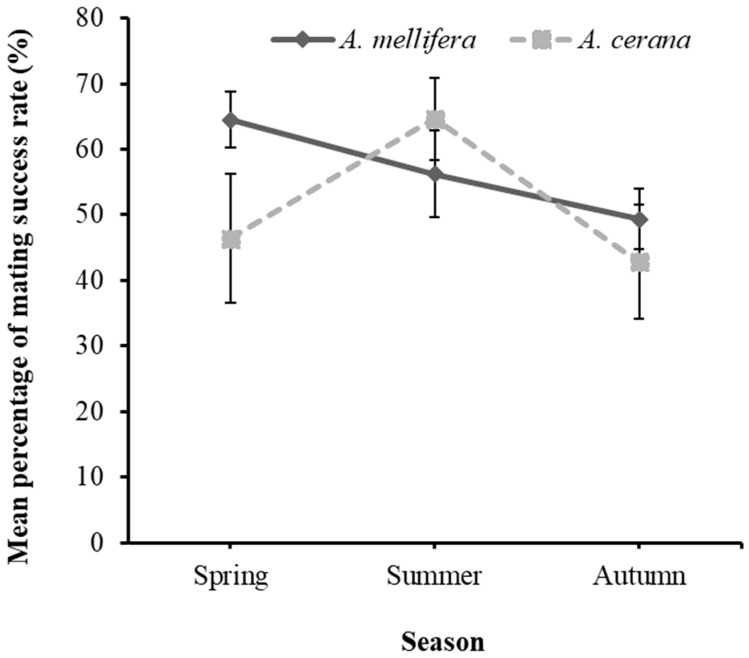
Seasonal variation in the mating success rate percentage.

The percentage of the mating success rate was recorded in spring, summer, and autumn in three consecutive years (2021–2023) (Figure 3).

**Figure 4:**
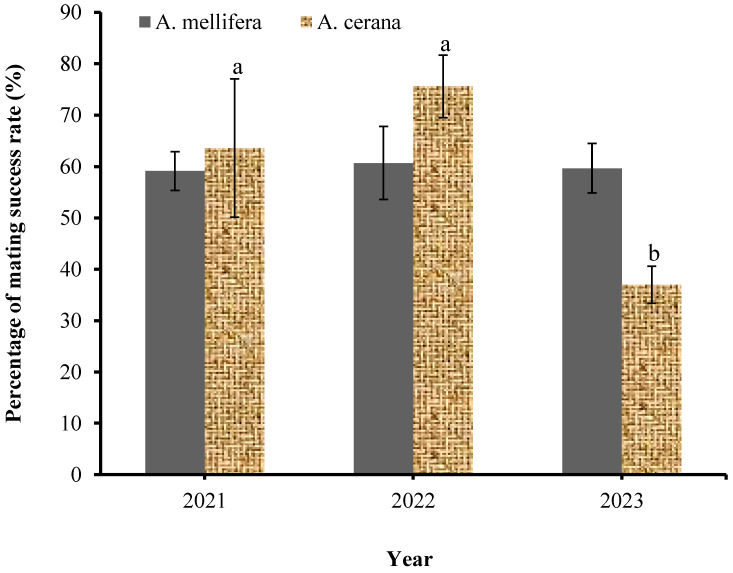
Annual variation in the mating success rate percentage. Error bars represent mean ± SE. Means with different small letters indicate a significant difference between at *p* < 0.05, one-way ANOVA, Tukey’s (HSD) post hoc test.

The mean percentage of the mating success rate in spring, summer, and autumn did not differ significantly for *A. mellifera* (F_2,31_ = 1.632, *p* = 0.212) and *A. cerana* (F_2,21_ = 2.105, *p* = 0.147). Although no significant difference was observed for the mean percentage of the mating success rate in spring, summer, and autumn, the mating success rate was the highest in spring and summer for *A. mellifera* and *A. cerana* (Figure 3). In *A. mellifera*, the mating success rate decreased from spring to autumn, while in *A. cerana*, the mating success rate increased from spring to summer and decreased in autumn. In order to have an insight on whether the climatic condition within the three years of breeding could affect mating success, we compared the mating success rate between the three years (Figure 4).

The mean percentage of the mating success rate in 2021, 2022, and 2023 did not differ significantly for *A. mellifera* (F_2,31_ = 0.020, *p* = 0.981) but differed significantly for *A. cerana* (F_2,21_ = 10.826, *p* < 0.001) in 2023 (Figure 4). The highest percentage of the mating success rate was recorded in 2022, while the lowest was recorded in 2023 for both *A. mellifera* and *A. cerana*, although it was insignificant for *A. mellifera* (Figure 4). 

### 3.3. Comparison of the Mating Success Rate of Honey Bees at Different Mating Stations

The mating success rate of honey bees was recorded at different mating stations during the breeding periods of 2021–2023 (Table 2).

**Figure 5:**
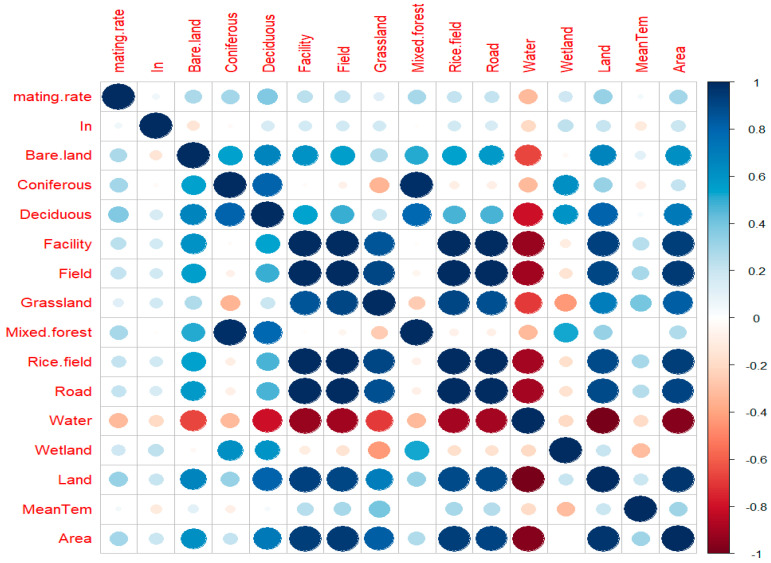
Correlation analysis between mating success rate and environmental factors within 2 km radius of land use coverage.

The mating success rate of honey bees differed significantly among the mating stations for both *A. cerana* (K = 20.525, df = 3, *p* = 0.0001) and *A. mellifera* (K = 13.213, df = 4, *p* = 0.01) (Table 2). The main source of variation in the mating success rate was identified in the mainland station (Wanju). In *A. cerana*, the island mating success rate was the highest in Wido Island compared to Nakwoldo and Sapsido Islands, though the difference was not significant compared to Nakwoldo Island (Table 2). The lowest mating success rate in *A. cerana* was recorded in Sapsido Island, while the highest mating success rate was recorded in Wanju Island (Table 2). In *A. mellifera*, the island mating success rate was the highest in Wido Island, while the lowest mating success rate was recorded in Wangdeungdo Island (Table 2). According to the results obtained, Wido Island stands as a favorable mating station for both *A. cerana* and *A. mellifera*. A correlation analysis was performed to assess the relationship between the mating success rate and various other factors (Figure 5). 

Water and wetland accounted for more than 70% of the total land cover in the breeding stations on the islands. The mating success rate increased relatively with the increased proportion of bare land and tended to decrease with an increasing proportion of water and wetland (Figure 5). Although not statistically significant, other factors showed a positive relationship with the mating success rate (Figure 5).

### 3.4. Variation in the Mating Success Rate in the Mainland and Island

**Figure 6:**
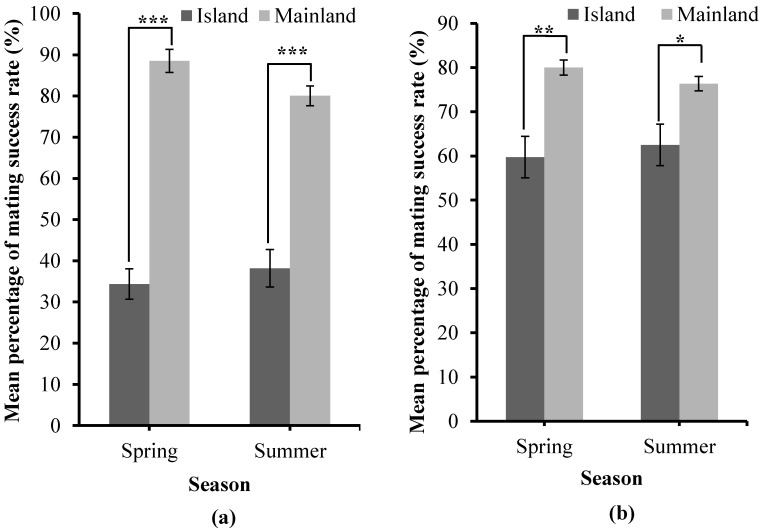
Mean percentage of mating success rate in the islands and mainland. (**a**) *A. cerana*. (**b**) *A. mellifera.* Significant difference: *** p < 0.0001, ** p < 0.01, * p < 0.05. Two-tailed Student’s *t*-test.

The mating success rates of honey bees bred in the mating stations on islands and mainland apiaries were recorded for both *A. mellifera* and *A. cerana* in spring and summer 2023 (Figure 6).

The mean percentage of the mating success rate on the islands and mainland differed significantly in spring (t = 2.160, *p* < 0.0001) and summer (t = 2.131, *p* < 0.0001) for *A. cerana* (Figure 6a). Similarly, the mean percentage of the mating success rate on the islands and mainland differs significantly in spring (t = 2.145, *p* = 0.001) and summer (t = 2.160, *p* = 0.011) for *A. mellifera* (Figure 6b). The percentage of the mating success rate did not differ significantly in spring and summer for island and mainland breeding in both *A. mellifera* and *A. cerana*. However, for *A. cerana*, on the islands, the mating success rate percentage was higher in the summer than in the spring; on the mainland, the mating success rate percentage was higher in the spring than in the summer (Figure 6a). In *A. mellifera*, the mating success rate was higher in the spring than in the summer for both island and mainland mating (Figure 6b). Generally, the mating success rate percentage was higher on the mainland compared to the islands for both *A. cerana* and *A. mellifera* (Figure 6).

### 3.5. Comparison of the Mating Success Rate between Purebred and Crossbred Lines of Honey Bees

**Figure 7:**
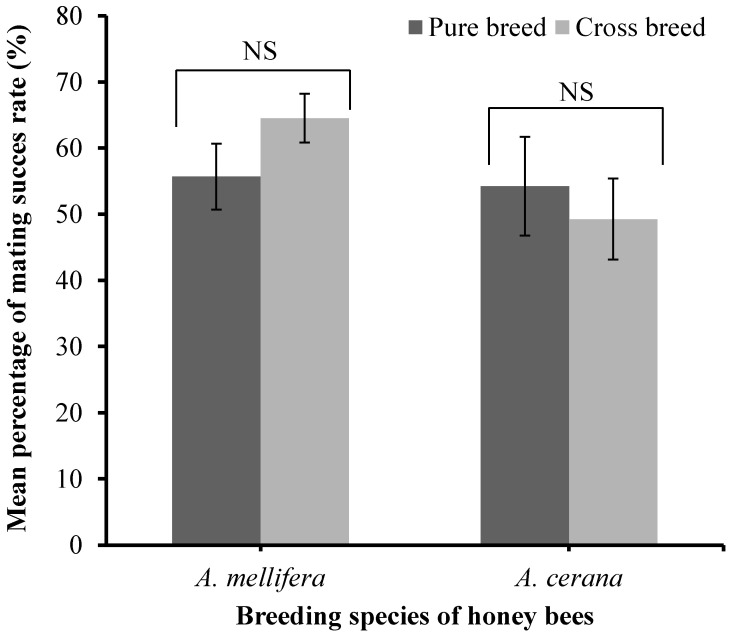
Mating success rate among pure breed and cross breed lines of *A. mellifera* and *A. cerana.* NS. Significant difference. Two-tailed Student’s *t*-test.

The mating success rate of purebred and crossbred lines of honey bees was recorded for both *A. cerana* and *A. mellifera* in the island mating stations (Figure 7).

The mating success rate of honey bees did not show any significant difference between the purebred and crossbred lines for both *A. mellifera* (t = 2.037, *p* = 0.172) and *A. cerana* (t = 2.074, *p =* 0.631) (Figure 7). The source of variation in the mating success rate of honey bees in the island and mainland mating stations could not be attributed to pure-breeding or crossbreeding. However, in *A. mellifera*, the difference between the mating success rate percentage for pure-breeding and cross breeding was higher than in *A. cerana* (Figure 7).

## 4. Discussion

In the modern era, according to Plates et al. [26], controlled mating does not only represent a personal advantage for individual breeders but it is also important for the genetic progress of the passive population of bees. In comparison with other agricultural species, controlled mating in honeybees appears to be hard to achieve. Environmental factors (landscape, weather, climate) are observed as the key components associated with mating success in honey bees. Weather conditions and predation may lead to 10% to 20% of queen bee losses during nuptial flights [49,50]. These factors are thought to vary with season and location. The results of this study demonstrated no significant variation in the mating success rate across different seasons. However, higher mating success rates were recorded in the spring and summer at higher temperatures and in good weather conditions. In a related study, the number of nuptial flights, which is a determinant of mating success in honey bees, was observed to vary between locations and was influenced by daily temperatures, which vary per season [21]. 

In contrast, it was reported that queens have no control over the number of times they mate and that the high mating frequencies of honey bees are simply a stochastic by-product of mating behavior and mate availability [51]. Studies have reported that the mating behavior of honey bee queens is influenced by environmental conditions, such as temperature, wind, and cloud cover [20,52,53]. Moreover, the low mating success rate observed in early autumn could be attributed to high wind speeds and ocean currents that hindered mating in the mating stations on the islands. Despite many flights reported for queens on islands [52,53], queens and drones take nuptial flights between 12:00 and 17:00 h, with maximum flight activity taking place between 13:00 and 16:00 h [54,55,56]. According to the National Oceanic and Atmospheric Administration [57], this period is valid for areas with high winds that are near a sea. This could hinder the flight activity of queen and drone bees, and consequently hinder mating success. High wind velocities attributed to poor weather conditions are known to reduce the mating success in honey bees. It was reported that winds over 3.9 m/s impaired mating [20,58] and that good weather resulted in 82–100% success rates for queen mating, while bad weather led to 59% success rates [59]. Relatively, we recorded a low mating success rate in Sapsido and Wangdeungdo Islands, which had more water cover compared to other islands. 

Previous studies have demonstrated that despite favorable temperatures and good weather at the end of August towards September, a low mating success rate of only 35% was linked to decreased nectar availability [59]. Our results are in accordance with those of other studies in that the mating success rate of honey bees is higher in the spring and summer (nectar flow periods) compared to autumn (nectar dearth period). Etelvina et al. [60] reported that the status of nectar flow had a significant effect on mating success and that mating success was higher during nectar flow (67%) season than periods of nectar dearth (52%). In our study, the mating success rate varied among mating stations for both *A. cerana* and *A. mellifera*. This could be attributed to the variations in resource availability as the highest percentage of mating success was recorded in Wido Island, which had a relatively high percentage of mixed forest and fields. It is thought that the availability of mixed forest and fields provided adequate floral vegetation that favored mating in this area, with the same scenario being observed for mainland mating. A low queen mating rate was reported for island populations compared to mainland populations of the same honey bee subspecies, *A. mellifera carnica* [61]. In this study, we recorded significant variations in the mating success rate percentage, which was lower on the islands compared to the mainland, in both honeybee subspecies (*A. cerana* and *A. mellifera*). The results of the landscape analysis demonstrate a distinct environmental differences between the island and mainland areas, with the islands being dominated by wetlands. The low mating success rate on the islands compared to the mainland could be attributed to the weather conditions of the islands, which may have influenced the duration of mating flights and consequently the number of drones that mated with queen bees. Woyke [62] reported that the ability of drones to mate one after the other with no complications affects the duration of mating flights. In another study conducted on Yemeni honeybee queens, Alattal et al. [35] suggested that the mating success rate could be affected by the ambient temperatures during sexual development and mating flights. Koeniger et al. [63] reported that most drones may limit their mating flight distances as a strategy to maximize the amount of time spent at drone congregation areas to increase their mating opportunities. However, this study did not investigate the time spent by drones at drone congregation areas. Despite this, we hypothesized that due to the poor weather conditions of the islands, the time spent by drones at drone congregation areas could be longer on the islands compared to the mainland.

The conservation of desired traits of particular honey bee subspecies without crossbreeding is important. For instance, it was reported that to prevent the risk of crossbreeding between subspecies, controlled mating is fundamental [64,65]. In this study, we recorded no variations in the mating success rate of pure- and crossbred lines in both *A. mellifera* and *A. cerana*. No uncontrolled mating occurred [66] under our controlled mating system. However, in some cases, queens can mate with drones of different subspecies, resulting in colonies of mixed subspecies [67]. In this study, there was no chance for queens to mate with drones from a different breed because of the mating stations being highly isolated from each other and the mainland. In honey bees, the conservation of genetic traits relies on reproductive fitness, which is an indicator of life-history traits, which are passed on from generation to generation. For instance, Keil and Sachser [68] reported that reproductive fitness is gained via polyandry in small mammals.

## 5. Conclusions

Genetic selection in honey bees is of particular interest to beekeepers and has been shown to be heritable [69,70,71]. However, the conservation and improvement of selected populations remain a challenge in many beekeeping operations due to their complex mating systems. The present study demonstrates the possible conditions for improving the mating success rate of honey bees bred in controlled systems (e.g., islands), with higher breeding success rates seen in mainland mating systems. It appears that islands with a relatively high percentage of deciduous and mixed forests, fields, and low percentage of wetland can play a potential role in the conservation and maintenance of a large population of honey bees through controlled breeding. Most importantly, this study depicts the variability in the mating success rate with respect to seasons and proposes that a large number of breeding populations can be achieved during the nectar flow season; however, this depends on the selected breeding site. This study has crucial practical applications for selecting reliable controlled systems for conserving and improving genetic resources in honey bees. 

## Figures and Tables

**Table 1:** Weather condition of mating stations.

Mating Stations	Weather Factors/Year								
	Average Temperature (°C)			Total Precipitation (mm)			Average Relative Humidity (%)		
	2021	2022	2023	2021	2022	2023	2021	2022	2023
Wido	14	13.5	14.3	1354	971.2	1853.6	76.3	73	74.8
Wangdeungdo	14	13.5	14.3	1354	971.2	1853.6	76.3	73	74.8
Sikdo	14	13.5	14.3	1354	971.2	1853.6	76.3	73	74.8
Nagwaldo	14	13.4	14.1	1202.6	802.7	1496.1	73.8	72	74.3
Sapsido	13.8	13.1	13.9	1107.9	1348.4	1726.7	71.6	70.3	71.8
Wanju	14.6	14	14.8	1496.6	1071.5	1986.6	68.4	66.2	68.2

Source: Meteorological Administration of Korea, 2023.

**Table 2:** Comparison of the mating success rate percentage of *A. cerana* and *A. mellefera* in different mating stations.

Breeding Stations		Mating Success Rate (%)	
		*A. cerana*	*A. mellifera*
Island	Wido	59.59 ± 6.2 b	61.25 ± 9.44 ab
	Wangdeungdo	-	57.26 ± 11.44 b
	Nakwoldo	45.11 ± 10.23 bc	60.27 ± 6.76 ab
	Sikdo	-	60.26 ± 4.03 b
	Sapsido	29.58 ± 1.72 c	-
Mainland	Wanju	84.28 ± 2.10 a	78.17 ± 1.22 a

In a column, means with different small letters differ significantly among mating stations; *p* < 0.05, Kruskal–Wallis test, Dunn’s procedure. Means are expressed as the mean ± SE of the mean.

## Data Availability

The data presented in this study are presented in this manuscript and are available upon request from the corresponding author.

## Supplementary Materials

The following supporting information can be downloaded at: https://www.mdpi.com/article/10.3390/biology13060444/s1, Figure S1: Schematic representation of breeding lines at mating stations; Figure S2: Placement of mating hives at mating stations: (a) island mating station and (b) mainland mating station.
