# Supplementary material for: The Influence of Environmental Factors on Site Selection Augment Breeding Success in Honey Bees: An Insight of Honey Bee Genetic Resource Conservation
Source: Biology (Basel). 2024 Jun 18;13(6):444. doi: 10.3390/biology13060444 (PMC11201093; doi:10.3390/biology13060444)
Supplement: Supplementary file 1 [file biology-13-00444-s001.zip › biology-3020466-supplementary.pdf]

SUPPLEMENTARY MATERIALS

The Influence of Environmental Factors on Site Selection Augment Breeding Success in Honey Bees: An Insight of Honey Bee Genetic Resource Conservation

Peter Njukang Akongte[1,2], Bo-Sun Park[1],Minwoong Son[1], Chang-hoon Lee[1], Daegeun Oh[1], Yong-Soo Choi[1],Dong-Won Kim[1]*

[1]Department of Agricultural Biology, National Institute of Agricultural Science, Wanju 55365, Republic of Korea

[2]Institute of Agricultural Research for Development (IRAD), Buea PMB 25, Cameroon

*Correspondence: dongwonkim@korea.kr

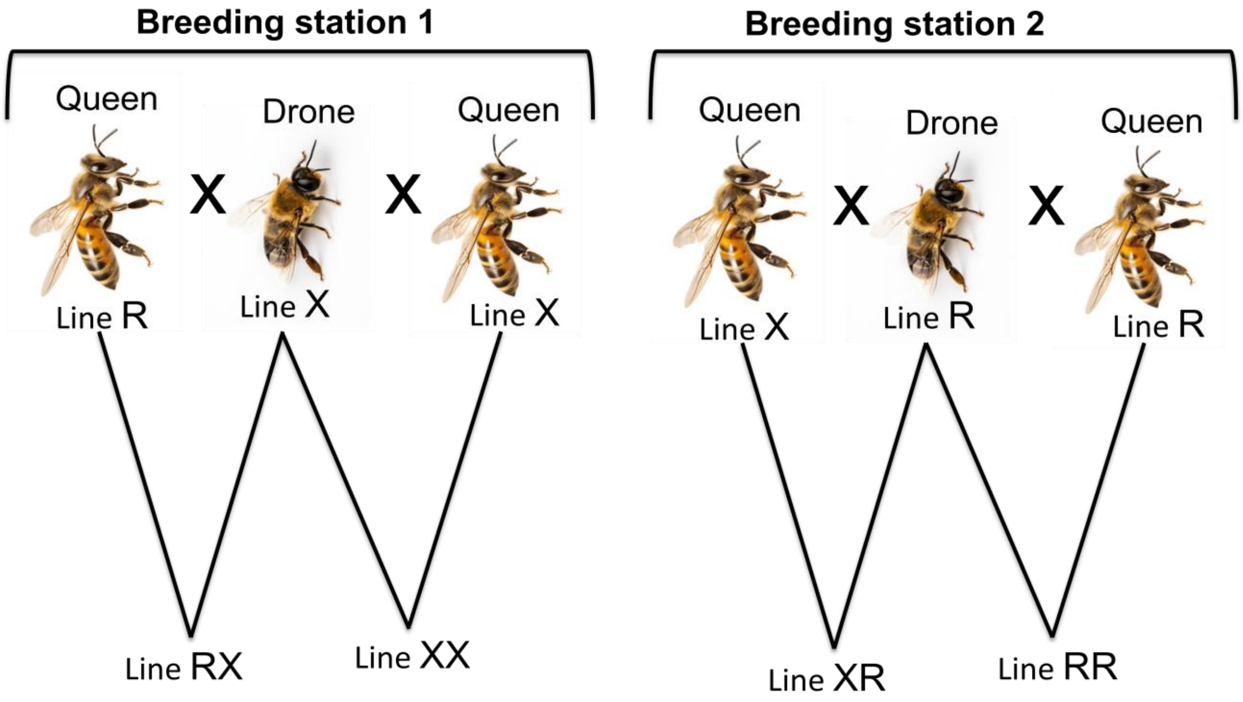

Figure S1: Schematic representation of breeding lines at mating stations.

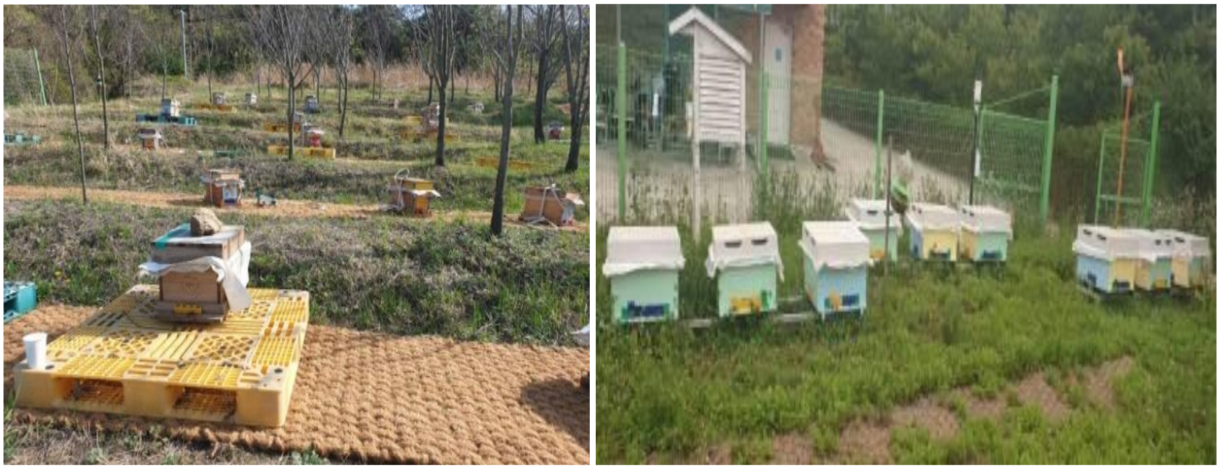

(a) (b)

Figure S2: Placement of mating hives at mating stations: island mating station (**a**) and mainland mating station (**b**).
